# SARS-CoV-2 testing and COVID-19–related primary care use among people with citizenship, permanent residency, and temporary immigration status: an analysis of population-based administrative data in British Columbia

**DOI:** 10.17269/s41997-023-00761-w

**Published:** 2023-04-04

**Authors:** Mei-ling Wiedmeyer, Shira Goldenberg, Sandra Peterson, Susitha Wanigaratne, Stefanie Machado, Elmira Tayyar, Melissa Braschel, Ruth Carrillo, Cecilia Sierra-Heredia, Germaine Tuyisenge, M. Ruth Lavergne

**Affiliations:** 1 Department of Family Practice, University of British Columbia, Vancouver, BC Canada; 2 Centre for Gender and Sexual Health Equity, Vancouver, BC Canada; 3 School of Public Health, San Diego State University, San Diego, CA USA; 4 Centre for Health Services and Policy Research, University of British Columbia, Vancouver, BC Canada; 5 ICES, Toronto, ON Canada; 6 Edwin H.S. Leong Centre, University of Toronto, Toronto, ON Canada; 7 Faculty of Health Sciences, Simon Fraser University, Burnaby, BC Canada; 8 Department of Family Medicine, Faculty of Medicine, Dalhousie University, Halifax, NS Canada

**Keywords:** Immigration, Migrants, Health services accessibility, Social determinants of health, COVID-19, Immigration, migrants, accessibilité des services de santé, déterminants sociaux de la santé, COVID-19

## Abstract

**Objectives:**

Having temporary immigration status affords limited rights, workplace protections, and access to services. There is not yet research data on impacts of the COVID-19 pandemic for people with temporary immigration status in Canada.

**Methods:**

We use linked administrative data to describe SARS-CoV-2 testing, positive tests, and COVID-19 primary care service use in British Columbia from January 1, 2020 to July 31, 2021, stratified by immigration status (citizen, permanent resident, temporary resident). We plot the rates of people tested and confirmed positive for COVID-19 by week from April 19, 2020 to July 31, 2021 across immigration groups. We use logistic regression to estimate adjusted odds ratios of a positive SARS-CoV-2 test, access to testing, and primary care among people with temporary status or permanent residency, compared with people who hold citizenship.

**Results:**

A total of 4,146,593 people with citizenship, 914,089 people with permanent residency, and 212,215 people with temporary status were included. Among people with temporary status, 52.1% had “male” administrative sex and 74.4% were ages 20–39, compared with 50.1% and 24.4% respectively among those with citizenship. Of people with temporary status, 4.9% tested positive for SARS-CoV-2 over this period, compared with 4.0% among people with permanent residency and 2.1% among people with citizenship. Adjusted odds of a positive SARS-CoV-2 test among people with temporary status were almost 50% higher (aOR 1.42, 95% CI 1.39, 1.45), despite having half the odds of access to testing (aOR 0.53, 95% CI 0.53, 0.54) and primary care (aOR 0.50, 95% CI 0.49, 0.52).

**Conclusion:**

Interwoven immigration, health, and occupational policies place people with temporary status in circumstances of precarity and higher health risk. Reducing precarity accompanying temporary status, including regularization pathways, and decoupling access to health care from immigration status can address health inequities.

## Résumé

**Objectifs:**

Le statut d’immigration temporaire confère des droits, des mesures de protection au travail et un accès aux services limités. Il n’y a pas encore de données de recherche sur les impacts de la pandémie de COVID-19 chez les personnes ayant un statut d’immigration temporaire au Canada.

**Méthode:**

Nous utilisons des données administratives maillées pour décrire le dépistage du SRAS-CoV-2, les tests positifs et l’utilisation des services de soins de première ligne liés à la COVID-19 en Colombie-Britannique entre le 1er janvier 2020 et le 31 juillet 2021, stratifiées selon le statut d’immigration (citoyenneté, résidence permanente, résidence temporaire). Nous reportons sur des graphiques les taux hebdomadaires de personnes testées et confirmées positives pour la COVID-19 entre le 19 avril 2020 et le 31 juillet 2021 dans les groupes d’immigration. Nous utilisons la régression logistique pour estimer les rapports de cotes ajustés d’un test positif pour le SRAS-CoV-2, de l’accès au dépistage et de l’accès aux soins primaires chez les personnes ayant le statut de résidents temporaires ou permanents comparativement aux personnes ayant la citoyenneté canadienne.

**Résultats:**

En tout, 4 146 593 citoyens, 914 089 résidents permanents et 212 215 résidents temporaires ont été inclus. Chez les personnes ayant le statut de résidents temporaires, 52,1 % étaient de sexe administratif « masculin » et 74,4 % avaient entre 20 et 39 ans, contre 50,1 % et 24,4 % respectivement chez les personnes ayant la citoyenneté. Chez les résidents temporaires, 4,9 % avaient obtenu un test positif pour le SRAS-CoV-2 au cours de la période de l’étude, contre 4 % chez les résidents permanents et 2,1 % chez les citoyens. La probabilité ajustée d’un test positif pour le SRAS-CoV-2 chez les personnes ayant le statut de résidents temporaires était près de 50 % plus élevée (RCa 1,42, IC de 95 % 1,39, 1,45), même si leurs probabilités d’accès au dépistage (RCa 0,53, IC de 95 % 0,53, 0,54) et aux soins primaires (RCa 0,50, IC de 95 % 0,49, 0,52) étaient moitié moindres.

**Conclusion:**

La conjugaison des politiques d’immigration, de santé et de main-d’œuvre met les personnes ayant le statut de résidents temporaires en situation de précarité et de risques accrus pour la santé. La réduction de la précarité qui accompagne le statut temporaire, dont les voies de régularisation, et le découplage entre l’accès aux soins de santé et le statut d’immigration pourraient répondre aux iniquités en santé.

## Introduction

Globally, uneven responses to the COVID-19 pandemic have caused disproportionate harm to immigrants, racialized people, and other socially and economically marginalized groups (Guttmann et al., 2020; Public Health Agency of Canada, 2021). COVID-19 outbreaks in places employing immigrants have been widely reported (Yang & Mojtehedzadeh, 2021) and suggest that many people with temporary immigration status have been placed in high-risk circumstances (Mojtehedzadeh, 2021), though research data are limited. Other people with temporary immigration status, such as international students, have reported serious impacts but are rarely included in government support measures or research (Firang, 2020). Published data show that refugee claimants in Canada experienced a high burden of COVID-19 (Redditt et al., 2020), while opportunities to claim asylum for people hoping to enter the country were curtailed due to pandemic-related travel restrictions (Edmonds & Flahault, 2021). An Ontario report in fall 2020 found that while immigrants, refugees, and other newcomers made up just 25% of the Ontario population, they experienced 43.5% of all COVID-19 cases (Guttmann et al., 2020). These initial findings indicate a relationship between immigration status and circumstances of vulnerability to COVID-19.

Canada regulates immigration through a variety of pathways which can be broadly grouped into temporary or permanent resident status, where permanent residents may become citizens after meeting requirements (Box 1). The number of temporary permits issued for work or study in Canada has increased dramatically over the past decade (Otero & Preibisch, 2015). Having temporary immigration status affords limited rights, workplace protections, and access to services which together create precarity—a multidimensional insecurity of work, residence, entitlements, and health (Goldring et al., 2009). This precarity produces harmful health outcomes in people with temporary immigration status in Canada (Cloos et al., 2020; Vahabi et al., 2018). We do not yet have research data on the health impact of the COVID-19 pandemic for people with temporary immigration status in Canada, nor data by immigration status outside of Ontario. We use health system data to describe SARS-CoV-2 testing and access to COVID-19–related primary care by immigration status for people eligible for the provincial insurance plan in the province of British Columbia.

## Methods

### Study setting

The borders of the province of British Columbia (BC) were defined on the lands of more than 200 Indigenous nations through resisted historical and ongoing colonial processes including forced displacement and internal migration (Claxton et al., 2021). The federal ministry Immigration, Refugees and Citizenship Canada (IRCC) controls movement across federal borders by issuing travel documents and screening potential permanent and temporary residents (Immigration and Refugee Protection Act, 2001), and determining admission as temporary or permanent residents. The province of BC is most densely populated in the lower mainland (the area surrounding Vancouver) which includes parts of Vancouver Coastal and Fraser health authorities. Over 80% of immigrants in BC live in these two health authorities, compared with just over 50% of people who hold citizenship (refer to Table 1 of data reported herein). BC is divided into eight economic development regions which include key industries that rely on temporary immigrants: Service (accommodation, food, retail), Construction and Manufacturing, Health Care and Social Assistance, and Natural Resources (agriculture, forestry, mining) (Lu, 2020; Province of British Columbia, 2022).

**Table 1:** Characteristics of people registered for MSP (Medical Services Plan of BC) who hold citizenship, permanent residency, and temporary status in British Columbia, January 2020 to July 2021, *N* (%)

	Citizenship	Permanent residency	Temporary status
Total number of people	4,146,593 (78.6)	914,089 (17.3)	212,215 (4.0)
Administrative sex*			
F	2,070,832 (49.9)	490,227 (53.6)	101,579 (47.9)
M	2,075,661 (50.1)	423,861 (46.4)	110,636 (52.1)
Age group**			
0–19	888,225 (21.4)	69,124 (7.6)	30,420 (14.3)
20–39	1,010,046 (24.4)	299,794 (32.8)	157,899 (74.4)
40–59	1,024,528 (24.7)	337,746 (36.9)	22,847 (10.8)
60 +	1,223,793 (29.5)	207,425 (22.7)	1049 (0.5)
Mean age (SD)	42.8 (24.2)	45.6 (18.4)	27.4 (9.4)
Neighbourhood income quintile			
Lowest	765,757 (18.5)	216,854 (23.7)	56,237 (26.5)
2nd	779,535 (18.8)	205,271 (22.5)	50,335 (23.7)
Middle	829,954 (20.0)	182,241 (19.9)	42,859 (20.2)
4th	878,632 (21.2)	157,811 (17.3)	29,196 (13.8)
Highest	813,501 (19.6)	138,289 (15.1)	25,193 (11.9)
Missing	79,214 (1.9)	13,623 (1.5)	8395 (4.0)
Rurality			
Metropolitan	2,591,249 (62.5)	822,735 (90.0)	185,817 (87.6)
Small urban	934,157 (22.5)	54,603 (6.0)	17,157 (8.1)
Rural	584,785 (14.1)	32,577 (3.6)	6313 (3.0)
Missing	36,402 (0.9)	4174 (0.5)	2928 (1.4)
Health authority			
Interior	790,147 (19.1)	45,242 (4.9)	16,048 (7.6)
Fraser	1,396,969 (33.7)	460,658 (50.4)	100,363 (47.3)
Vancouver Coastal	849,800 (20.5)	328,578 (35.9)	72,107 (34.0)
Island	796,718 (19.2)	62,072 (6.8)	15,679 (7.4)
Northern	281,425 (6.8)	15,024 (1.6)	5439 (2.6)
Missing	31,534 (0.8)	2515 (0.3)	2579 (1.2)
Mean days registered for MSP within study period (SD)***	447.3 (47.5)	447.8 (44.6)	355.8 (120.5)

*100 people with citizenship and < 5 people with permanent residency had unknown administrative sex, **< 5 people with citizenship were missing age data

***The maximum is 456 (366 in 2020 and 90 in 2021, 2020 was a leap year)

The Medical Services Plan (MSP) is BC’s provincial health insurance program that covers health care benefits for eligible BC residents. People who hold Canadian citizenship and people with permanent residency are eligible for MSP provided they meet some conditions (Government of British Columbia, n.d.). Some people who hold temporary study or work permits that are valid for six or more months are also eligible, including refugee claimants and convention refugees who also hold work permits (Government of British Columbia, n.d.). In April 2020, MSP extended temporary coverage for some people with expired work or study permits and established a mechanism to reimburse providers for COVID-19–related care for people without MSP, but not other health services (Ministry of Health, 2021). Usually, people who are not eligible, including those whose temporary permits expire, have no access to public health insurance in BC. New eligible residents in BC are required to undergo a 3-month wait period prior to health insurance activation. The province temporarily eliminated this policy in March 2020, only to reinstate it in August 2020 (Kines, 2020).

### Data and study population

We accessed linked, population-based administrative data through Population Data BC, including the MSP registry file (British Columbia Ministry of Health, 2021a), SARS-CoV-2 testing (British Columbia Ministry of Health, 2021c), and physician payments (British Columbia Ministry of Health, 2021b). Population Data BC (PopData) is a multi-university resource that coordinates applications to access and linkage of administrative data from multiple government partners. Data are securely linked using both deterministic and probabilistic linkage with only study-specific unique identifiers supplied to the research team (https://www.popdata.bc.ca/datalinkage/process). Only approved analysts access these data within a secure research environment and we only report statistics about groups, so results cannot be linked to individual people. Requests to access the data sets used for this study can be made to PopData (https://www.popdata.bc.ca/data_access). Ethics approval was obtained from the University of British Columbia and Simon Fraser University.

Testing and service use data cover the period from January 1, 2020 to July 31, 2021. Analyses included all people registered for MSP at any time January 1, 2020–March 31, 2021. People who registered in April, May, June, and July 2021 could not be included as a 3-month wait period after registration means they would not have accessed publicly funded services during the study period. We excluded people identified in the MSP registry as visitors, diplomats, and on working holiday visas in BC, as their usual place of residence is outside of Canada. Access to data provided by the data steward(s) is subject to approval but can be requested for research projects through the data steward(s) or their designated service providers. All inferences, opinions, and conclusions drawn in this publication are those of the author(s), and do not reflect the opinions or policies of the data steward(s).

### Study design

We report weekly rates of SARS-CoV-2 testing, positivity, and COVID-19 primary care service stratified by immigration status (citizen, permanent resident, temporary resident) over the period from April 19, 2020 to July 31, 2021. We then use logistic regression to estimate odds ratios of having one or more SARS-CoV-2 tests, positive SARS-CoV-2 tests, and primary care visits related to COVID-19 among people with temporary status or permanent residency, compared to people who hold citizenship, examining outcomes cross-sectionally over the entire study period from January 1, 2020 to July 31, 2021.

### Measures

Immigration status was collected from MSP registration data. People registering for MSP are required to provide documentation confirming eligible status in Canada. People with *citizenship* include both people born in Canada or to Canadian parents and people who immigrated and subsequently provided documentation confirming citizenship. People with *permanent residency* include economic and family class immigrants, as well as resettled refugees and successful asylum (Canadian Council for Refugees, 2010) or Humanitarian and Compassionate applicants. People with *temporary status* include people with work permits (including Temporary Foreign Workers) or study permits, refugee claimants, and convention refugees whose refugee claim was accepted but who do not yet have permanent residency. Where people held multiple statuses, we assigned them to the status held longest during the study period because it would better represent a person’s access to health care than the latest status held during that time period.

The MSP registration form contains a variable labelled “Gender” with the options “M” and “F” provided (presumed to be abbreviations of the sexes “male” and “female”). We refer to this variable as “administrative sex” and write “male” and “female” when describing this variable. Whether responses reflect gender, sex assigned at birth or legal sex cannot be determined. Age was calculated as of January 1, 2021. Neighbourhood income quintiles were determined based on census enumeration area of residence, assigned using the Postal Code Conversion File (PCCF +) (Wilkins, 2009). We used the Statistics Canada Statistical Area Classification Metropolitan Influences Zones (Statistics Canada, 2018) to group metropolitan areas (census metropolitan areas), small urban areas (census agglomerations), and rural/remote settings (areas with strong to no metropolitan influence). We also report regional health authority of residence and the number of days enrolled between January 2020 and March 2021.

We determined the proportion of people who received one or more SARS-CoV-2 tests and the proportion of people with one or more positive tests in BC within the study period from the SARS-CoV-2 testing file (Appendix Table 5). BC uses ICD9 coding for all billing and encounter submissions to MSP rather than ICD10 or 11. We used the C19 code for COVID-19 which reflects what care providers were instructed to use within their billing records. Access to any COVID-19–related primary care is defined as one or more outpatient visits (location as office, long-term care, home, or virtual) with a family doctor where the ICD9 diagnosis code submitted was C19 and/or where fee items specific to COVID-19 office visits (T13701 with test, T13702 without test) were billed to MSP. This outcome measure was intended to reflect publicly funded support in managing suspected or confirmed COVID-19 and also reflects accessibility of primary care services broadly. As such, we report the percentage for all people, not only those with a positive SARS-CoV-2 test. Finally, we plot the number of SARS-CoV-2 tests and the number of positive tests per 100,000 people per week over the study period. Where people had more than one test or positive tests within the week, we counted only one. The denominator for all analysis includes people enrolled at any time during the study period. We have data on the number of days enrolled in MSP within the study period, but not the dates coverage started or stopped, so we cannot determine who was enrolled within each week.

### Analysis

We report outcomes as numbers and percentages by immigration group, and stratified by administrative sex, age, income quintile, rurality, and health authority. We plotted the weekly number of individuals tested and the number of individuals confirmed positive for SARS-CoV-2 per 100,000 population from April 19, 2020 to July 31, 2021 across immigration groups. As these are population-based rates reflecting the entire population of interest and not a sample, we do not supply confidence intervals or otherwise characterize uncertainty.

We use logistic regression to estimate adjusted odds ratios of a positive SARS-CoV-2 test, access to testing, and COVID-19–related primary care among people with temporary status or permanent residency, compared to people who hold citizenship. Adjusted models include administrative sex, age group (categorical), neighbourhood income quintile, rurality of residence (metropolitan, small urban, rural, missing), and health authority of residence. These variables were selected as potential confounders as they are independent predictors of COVID-19 testing and services use and associated with immigration status. While the data set completely captures the outcomes of interest, data were missing for individual characteristics related to place of residence (neighbourhood income, urban/rural residence, health authority of residence) reflecting individuals with no accurate address information within their file. This may correspond to precarity of housing, which is likely predictive of outcomes, and may be associated with immigration. For this reason, we retain a “missing” category for these variables in regression analysis (Table 3). All analysis was completed in SAS 9.4.

## Results

Between January 2020 and March 2021, 4,146,593 people with citizenship, 914,089 people with permanent residency, and 212,215 people with temporary status registered for MSP (Table 1). The people with temporary status were composed of students (51%), workers (46%), and refugee claimants (3%) (Appendix Table 4). Among people with temporary status, 52.1% had “male” administrative sex compared to 46.4% of people with permanent residency and 50.1% of people with citizenship. Higher percentages of people with temporary status were in the 20–39-year-old age category (74.4% compared with 32.8% of people with permanent residency and 24.4% of people with citizenship), reflecting the younger ages of people with study and work permits who make up the temporary status group. Higher percentages of people with temporary status lived in the lowest income neighbourhoods (26.5%, followed by 23.7% with permanent residency and 18.5% with citizenship). The percentage of people living in metropolitan centres was higher among people with both temporary status (87.6%) and permanent residency (90.0%) compared to people with citizenship (62.5%). Almost half of people with temporary status (47.3%) and permanent residency (50.4%) lived in the Fraser Health Authority, compared with 33.7% of people with citizenship. Mean days registered for MSP within the 456-day study period were lower among people with temporary status (355.8 days) compared with people with permanent residency (447.8 days) or citizenship (447.3 days).

The percentage of people who ever received a SARS-CoV-2 test was lowest among people with temporary status (24.1%) followed by people with permanent residency (26.0%) and citizenship (28.1%) (Table 2). This pattern of lower testing among people with temporary status, followed by permanent residency, and then citizenship, was also observed across administrative sex, income quintile, and health authority, as well as among all age groups except 60 + and both urban and small urban settings. Those with permanent residency had a slightly lower percentage of SARS-CoV-2 testing than those with temporary status among people over age 60. Within rural/remote settings, a slightly higher percentage of people with temporary status received a SARS-CoV-2 test (21.6%) compared with people with permanent residency (20.6%) and citizenship (20.8%).

**Table 2:** COVID-19 testing, infection, and primary care use among people registered for MSP who hold citizenship, permanent residency, and temporary status in British Columbia, January 2020 to July 2021, *N* (%)

	Had one or more SARS-CoV-2 tests			Had one or more SARS-CoV-2 positive results			Had one or more COVID-19 primary care visits		
	Citizenship	Permanent residency	Temporary status	Citizenship	Permanent residency	Temporary status	Citizenship	Permanent residency	Temporary status
Total	1,163,694 (28.1)	237,363 (26.0)	51,090 (24.1)	88,646 (2.1)	36,918 (4.0)	10,341 (4.9)	186,104 (4.5)	48,680 (5.3)	5612 (2.6)
Administrative sex									
F	624,407 (30.2)	131,253 (26.8)	24,970 (24.6)	43,459 (2.1)	18,957 (3.9)	4205 (4.1)	108,693 (5.2)	28,930 (5.9)	3034 (3.0)
M	539,285 (26.0)	106,110 (25.0)	26,120 (23.6)	45,187 (2.2)	17,961 (4.2)	6136 (5.5)	77,411 (3.7)	19,750 (4.7)	2578 (2.3)
Age group									
0–19	245,370 (27.6)	14,839 (21.5)	4127 (13.6)	19,395 (2.2)	2341 (3.4)	698 (2.3)	30,168 (3.4)	1850 (2.7)	356 (1.2)
20–39	370,491 (36.7)	97,504 (32.5)	41,883 (26.5)	30,994 (3.1)	14,260 (4.8)	8517 (5.4)	49,923 (4.9)	16,445 (5.5)	4397 (2.8)
40–59	292,280 (28.5)	86,994 (25.8)	4874 (21.3)	22,736 (2.2)	14,211 (4.2)	1095 (4.8)	53,344 (5.2)	19,998 (5.9)	827 (3.6)
60 +	255,553 (20.9)	38,026 (18.3)	206 (19.6)	15,521 (1.3)	6106 (2.9)	31 (3.0)	52,669 (4.3)	10,387 (5.0)	32 (3.1)
Neighbourhood income quintile									
Lowest	210,765 (27.5)	55,427 (25.6)	14,098 (25.1)	18,041 (2.4)	9606 (4.4)	2978 (5.3)	32,407 (4.2)	11,565 (5.3)	1564 (2.8)
2nd	216,203 (27.7)	56,738 (27.6)	13,295 (26.4)	17,531 (2.2)	10,332 (5.0)	3174 (6.3)	35,297 (4.5)	12,136 (5.9)	1439 (2.9)
Middle	236,279 (28.5)	48,504 (26.6)	10,255 (23.9)	18,198 (2.2)	7648 (4.2)	2110 (4.9)	39,020 (4.7)	10,451 (5.7)	1261 (2.9)
4th	258,496 (29.4)	41,866 (26.5)	6919 (23.7)	18,268 (2.1)	5511 (3.5)	1161 (4.0)	40,133 (4.6)	8094 (5.1)	714 (2.4)
Highest	227,807 (28.0)	32,145 (23.2)	5446 (21.6)	15,204 (1.9)	3519 (2.5)	814 (3.2)	36,976 (4.5)	5965 (4.3)	536 (2.1)
Missing	14,144 (17.9)	2683 (19.7)	1077 (12.8)	1404 (1.8)	302 (2.2)	104 (1.2)	2271 (2.9)	469 (3.4)	98 (1.2)
Rurality									
Metropolitan	811,802 (31.3)	218,053 (26.5)	46,431 (25.0)	68,214 (2.6)	35,114 (4.3)	9545 (5.1)	124,769 (4.8)	44,400 (5.4)	4908 (2.6)
Small urban	227,432 (24.3)	12,054 (22.1)	3097 (18.1)	13,109 (1.4)	1145 (2.1)	555 (3.2)	34,786 (3.7)	2209 (4.0)	320 (1.9)
Rural	121,657 (20.8)	6721 (20.6)	1363 (21.6)	7121 (1.2)	620 (1.9)	223 (3.5)	26,001 (4.4)	1972 (6.1)	358 (5.7)
Missing	2803 (7.7)	535 (12.8)	199 (6.8)	202 (0.6)	39 (0.9)	18 (0.6)	548 (1.5)	99 (2.4)	26 (0.9)
Health authority									
Interior	191,672 (24.3)	10,187 (22.5)	3279 (20.4)	11,310 (1.4)	963 (2.1)	485 (3.0)	24,588 (3.1)	1451 (3.2)	197 (1.2)
Fraser	482,286 (34.5)	140,318 (30.5)	27,877 (27.8)	46,582 (3.3)	26,719 (5.8)	7269 (7.2)	77,162 (5.5)	29,900 (6.5)	3172 (3.2)
Vancouver Coastal	257,065 (30.3)	73,428 (22.3)	16,651 (23.1)	20,219 (2.4)	8450 (2.6)	2205 (3.1)	43,601 (5.1)	14,815 (4.5)	1807 (2.5)
Island	176,678 (22.2)	10,818 (17.4)	2318 (14.8)	4482 (0.6)	384 (0.6)	146 (0.9)	26,918 (3.4)	1777 (2.9)	232 (1.5)
Northern	54,641 (19.4)	2451 (16.3)	835 (15.4)	5970 (2.1)	391 (2.6)	224 (4.1)	13,519 (4.8)	712 (4.7)	191 (3.5)
Missing	1352 (4.3)	161 (6.4)	130 (5.0)	83 (0.3)	11 (0.4)	12 (0.5)	316 (1.0)	25 (1.0)	13 (0.5)

The percentage of people with one or more positive SARS-CoV-2 tests was highest among people with temporary status (4.9%) followed by permanent residency (4.0%) and citizenship (2.1%). Differences in the percentages of people with one or more positive tests by immigration group were greater among people with “male” administrative sex, where 5.5% of people with temporary status had one or more positive tests, compared with 4.2% of people with permanent residency, and 2.2% of people with citizenship (Table 2). The pattern of higher percentages of people who tested positive for SARS-CoV-2 among people with temporary status, followed by permanent residency, and then citizenship was observed across all income quintiles and metropolitan, urban, and rural settings. Across the entire study population, people ages 20–39, in lower income neighbourhoods, and in metropolitan centres (especially Fraser Health Authority) had higher percentages of positive tests. Within these categories, people with temporary status still had the highest percentages of positive tests.

**Fig. 1:**
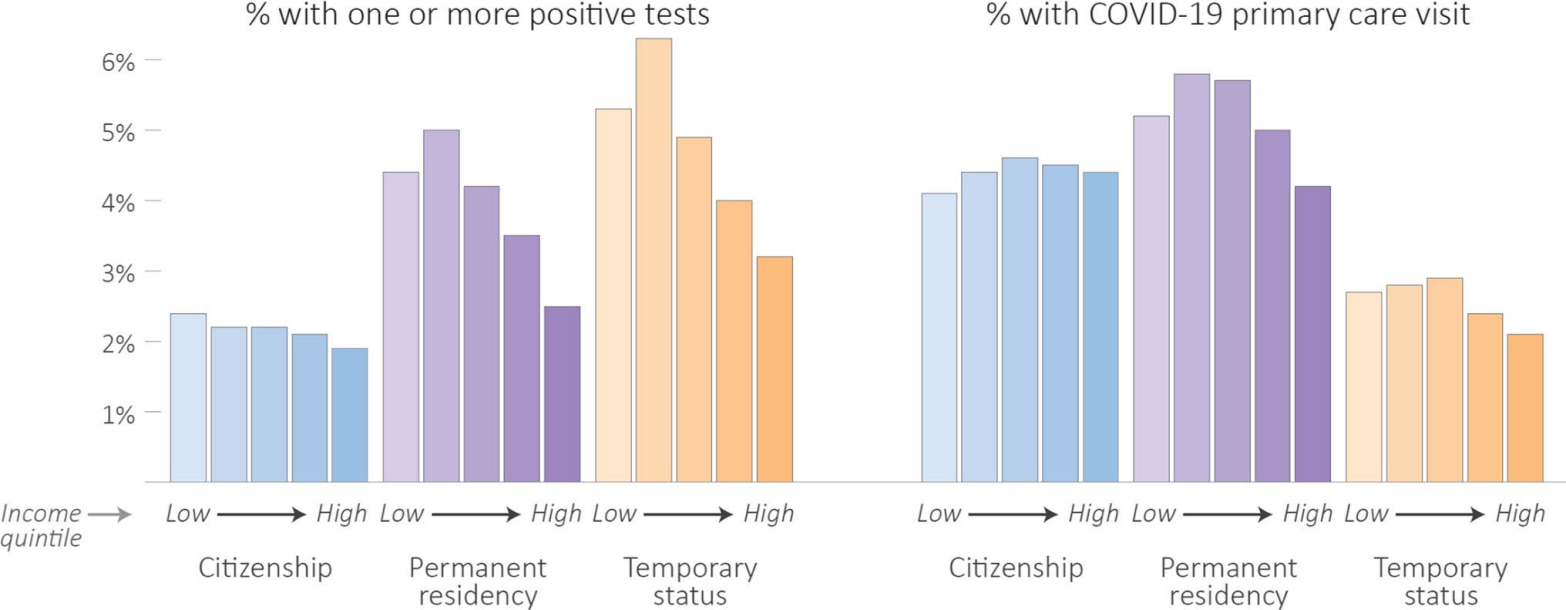
Percentages of positive tests and COVID-19 primary care visits by immigration status and neighbourhood income quintile

Despite being the group with the highest percentage of people with positive SARS-CoV-2 tests, people with temporary status had a lower percentage of COVID-19–related primary care visits (2.6%) than both people with permanent residency (5.2%) and citizenship (4.4%). Differences were particularly apparent by administrative sex. While the percentage of people with positive SARS-CoV-2 tests was highest among people with “male” administrative sex and temporary status (5.5%), access to primary care was lowest in this group (2.3%). Figure 1 shows the relationships between SARS-CoV-2 testing and COVID-19–related primary care, immigration status, and income. This describes a relationship between poverty and immigration status, in that there is a lower percentage of testing, higher percentage of positive tests, and lower percentage of primary care visits with decreasing neighbourhood incomes for all groups, though people with temporary immigration status experience the lowest testing, highest SARS-CoV-2 test positivity, and lowest percentage of primary care visits within each neighbourhood income quintile. This effect is such that people with temporary status who live in the highest income neighbourhoods still had higher test positivity than people with citizenship in the lowest income neighbourhoods.

Multivariable models confirm differences in testing, test positivity, and access to primary care (Table 3). Adjusted odds of a positive SARS-CoV-2 test among people with temporary immigration status were almost 50% higher (aOR 1.42, 95% CI 1.39, 1.45), despite having half the odds of access to testing (aOR 0.53, 95% CI 0.53, 0.54) and primary care (aOR 0.50, 95% CI 0.49, 0.52). As these models include variables reflecting income and location of residence which are themselves, at least in part, shaped by immigration status (especially for people with temporary status), the adjusted odds ratios we estimate may be somewhat biased toward the null, though still reflect significant and meaningful differential health outcomes.

**Table 3:** Unadjusted and adjusted odds of COVID-19 testing, infection, and primary care use among people registered for MSP who hold citizenship, permanent residency, and temporary status in British Columbia, January 2020 to July 2021, odds ratio (95% CI)

	Had one or more SARS-CoV-2 tests		Had one or more SARS-CoV-2 positive results		Had one or more COVID-19 primary care visits	
	Unadjusted	Adjusted	Unadjusted	Adjusted	Unadjusted	Adjusted
Immigration group (reference is people who hold citizenship)						
Permanent residency	0.90 (0.89, 0.90)	0.71 (0.70, 0.71)	1.93 (1.90, 1.95)	1.38 (1.36, 1.39)	1.20 (1.18, 1.21)	0.97 (0.96, 0.98)
Temporary status	0.81 (0.80, 0.82)	0.53 (0.53, 0.54)	2.34 (2.30, 2.39)	1.42 (1.39, 1.45)	0.58 (0.56, 0.59)	0.50 (0.49, 0.52)
Administrative sex (reference is F)						
M	0.83 (0.83, 0.84)	0.82 (0.82, 0.83)	1.06 (1.05, 1.07)	1.06 (1.05, 1.07)	0.71 (0.71, 0.72)	0.72 (0.71, 0.72)
Age group (reference is 0–19)						
20–39	1.47 (1.46, 1.48)	1.61 (1.60, 1.62)	1.65 (1.62, 1.67)	1.48 (1.46, 1.51)	1.51 (1.49, 1.53)	1.57 (1.55, 1.60)
40–59	1.07 (1.06, 1.08)	1.12 (1.11, 1.13)	1.23 (1.21, 1.25)	1.17 (1.15, 1.19)	1.69 (1.67, 1.72)	1.68 (1.66, 1.71)
60 +	0.72 (0.72, 0.73)	0.74 (0.73, 0.74)	0.67 (0.66, 0.68)	0.72 (0.71, 0.74)	1.38 (1.36, 1.40)	1.40 (1.38, 1.42)
Neighbourhood income quintile (reference is highest)						
Lowest	0.99 (0.98, 0.99)	0.97 (0.97, 0.98)	1.49 (1.46, 1.51)	1.33 (1.31, 1.36)	0.98 (0.97, 1.00)	0.98 (0.97, 0.99)
2nd	1.02 (1.02, 1.03)	0.99 (0.98, 1.00)	1.51 (1.48, 1.54)	1.33 (1.31, 1.36)	1.06 (1.05, 1.08)	1.05 (1.03, 1.06)
Middle	1.04 (1.03, 1.04)	0.99 (0.98, 1.00)	1.33 (1.31, 1.36)	1.19 (1.16, 1.21)	1.08 (1.07, 1.10)	1.06 (1.04, 1.07)
4th	1.09 (1.08, 1.09)	1.01 (1.00, 1.02)	1.17 (1.15, 1.20)	1.03 (1.01, 1.05)	1.03 (1.02, 1.05)	0.99 (0.98, 1.00)
Missing	0.53 (0.52, 0.54)	0.96 (0.94, 0.98)	0.83 (0.79, 0.87)	1.49 (1.41, 1.57)	0.57 (0.55, 0.60)	0.86 (0.82, 0.90)
Rurality (reference is metropolitan)						
Small urban	0.75 (0.75, 0.75)	0.96 (0.96, 0.97)	0.46 (0.46, 0.47)	0.77 (0.75, 0.79)	0.76 (0.75, 0.77)	1.06 (1.05, 1.08)
Rural	0.62 (0.61, 0.62)	0.81 (0.80, 0.81)	0.40 (0.39, 0.41)	0.68 (0.66, 0.70)	0.94 (0.93, 0.95)	1.41 (1.39, 1.43)
Missing	0.18 (0.17, 0.18)	0.92 (0.86, 0.97)	0.16 (0.14, 0.18)	0.56 (0.47, 0.66)	0.26 (0.24, 0.29)	1.17 (1.04, 1.32)
Health authority (reference is Vancouver Coastal)						
Interior	0.83 (0.83, 0.84)	0.86 (0.85, 0.87)	0.61 (0.59, 0.62)	0.86 (0.84, 0.88)	0.63 (0.62, 0.64)	0.54 (0.53, 0.55)
Fraser	1.30 (1.30, 1.31)	1.28 (1.28, 1.29)	1.70 (1.68, 1.73)	1.74 (1.72, 1.76)	1.18 (1.17, 1.20)	1.21 (1.20, 1.22)
Island	0.73 (0.73, 0.73)	0.72 (0.72, 0.73)	0.23 (0.22, 0.24)	0.30 (0.29, 0.31)	0.68 (0.67, 0.69)	0.63 (0.62, 0.64)
Northern	0.62 (0.62, 0.63)	0.62 (0.61, 0.62)	0.89 (0.86, 0.91)	1.32 (1.28, 1.37)	1.00 (0.98, 1.02)	0.86 (0.85, 0.88)
Missing	0.10 (0.10, 0.11)	0.12 (0.11, 0.13)	0.10 (0.08, 0.12)	0.16 (0.12, 0.20)	0.16 (0.15, 0.18)	0.20 (0.17, 0.24)

**Fig. 2:**
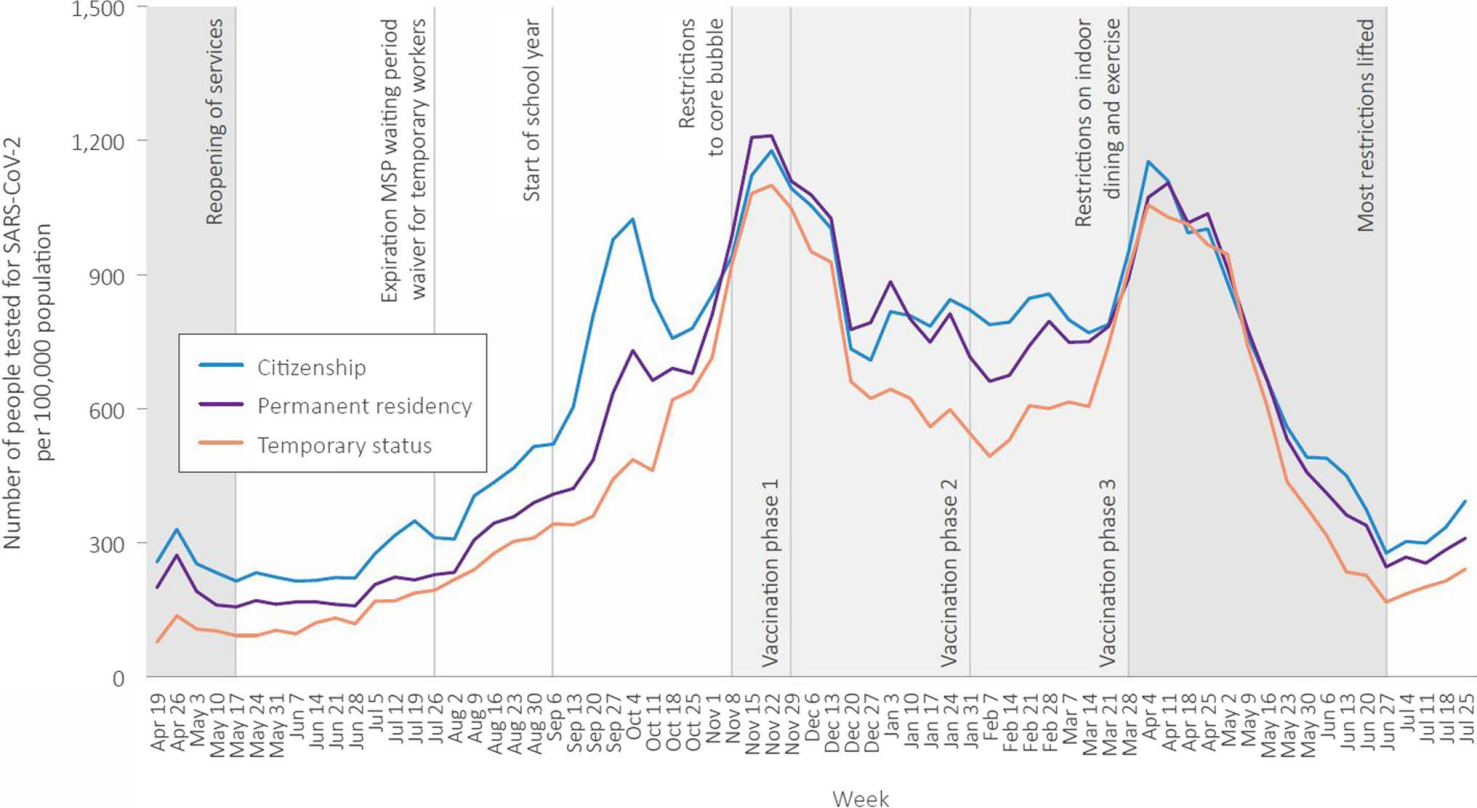
Number of people tested for SARS-CoV-2 per 100,000 population per week, by immigration status, April 2020 to July 2021

**Fig. 3:**
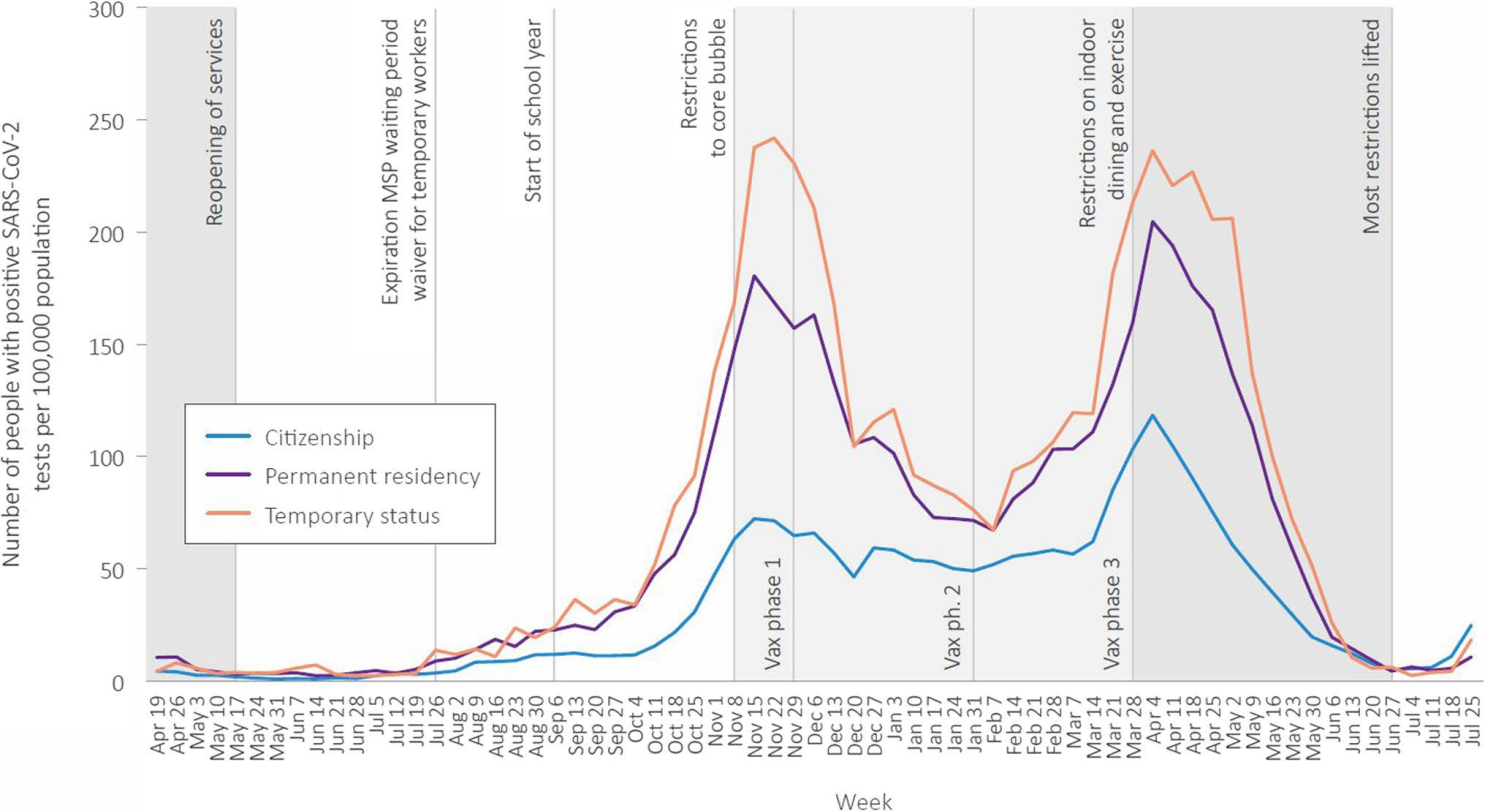
Number of people with positive SARS-CoV-2 tests per 100,000 population per week, by immigration status, April 2020 to July 2021

Examining patterns in testing over time shows that people with citizenship accessed more testing in the period preceding the second wave, and in between the second and third waves, though access to testing converged at the peaks of waves 2 and 3 (Fig. 2). The differences in positive tests between groups were apparent in both waves 2 and 3 (Fig. 3).

## Discussion

This study documents notable differences in both risk of COVID-19 infection and access to health care, finding that temporary immigration status corresponds to higher test positivity for SARS-CoV-2, but lower access to testing and COVID-19–related primary care. This pattern is persistent by administrative sex, age group, neighbourhood income quintile, health authority, and in both metropolitan and small urban settings, as well as in multivariable models. We interpret these differences as inequitable since they correspond to disadvantages associated with immigration class that are both preventable and amenable to correction through social policy.

More positive tests among people with “male” administrative sex who have temporary status or permanent residency, combined with lower access to primary care, may suggest gendered facets of COVID-19 risk management, though we cannot measure gender directly due to the way the health system collects these data. Similarly, lower testing rates but higher rates of laboratory-confirmed COVID-19 infection as well as delayed testing were observed among presumed males in Ontario (Joh et al., 2021; Stall et al., 2020). In our study, higher percentages of testing and visits for COVID-19–related primary care among people with temporary status in rural/remote settings may reflect targeted testing and outreach in agricultural workplaces where people in the Temporary Foreign Worker Program are employed. However, differences in test positivity observed in wave 2 and then wave 3 (Fig. 2) in all geographic regions suggest targeted workplace outreach was insufficient to address dramatic differences in exposure risk and access to health services shaped by temporary immigration status.

Previous reporting has identified social determinants of health such as poverty (Canadian Blood Services, 2021; Weill et al., 2020), poor housing conditions (Ahmad et al., 2020), occupational conditions (St‐Denis, 2020), language barriers (Siemaszko, 2020), systemic racism (Public Health Agency of Canada, 2021), and policing (Luscombe & McClelland, 2020) as placing people in circumstances of higher risk of COVID-19 transmission, morbidity, and mortality (Statistics Canada, 2020; Sundaram et al., 2021). People with temporary immigration status experience a convergence of some or all of these determinants, constructing a context of precarity with a profound health impact (Weiler et al., 2020) reaching beyond health insurance coverage. Though, for residents whose temporary immigration status ends and renders them uninsured, these health impacts are magnified (Caulford & Vali, 2006). This intersection of poverty and immigration status appears in our results, where there was increasing burden of COVID-19 with decreasing neighbourhood incomes for all groups; yet, people with temporary immigration status were both more likely to live in lower income neighbourhoods and also experience the highest SARS-CoV-2 test positivity in those neighbourhood income quintiles. People with temporary immigration status are also more likely than citizens or permanent residents to be racialized within Canada (Tuyisenge & Goldenberg, 2021); though, Indigenous people and Black communities with histories of enslavement have been here long before the border currently enforced under federal immigration policy, and also continue to struggle against a contested citizenship and inequitable health outcomes (Maynard, 2019; Turpel-Lafond, 2020). The interconnected effects of poverty, racism, and control of movement continue the history of settler colonialism and racial capitalism in the Canadian state formation, where colonial border regulations not only regulate and control movement of Indigenous people on their lands, but also categorize immigrants upon entry and specific, often racialized groups are placed in the temporary programs that can then determine employment, income, and geographic circumstances (Claxton et al. 2021). Canada’s immigration policy for temporary residents (both workers and students) constrains their movement and health while benefiting from their labour and economic contributions. This historic and ongoing asymmetry of benefit underlies the expansion of temporary immigration programs over the past four decades and is persistently challenged by temporary and precarious migrants advocating to uphold their human and labour rights (Fairey et al., 2008).

### Strengths and limitations of the study

This analysis has strengths and limitations related to the data available to study this topic. Due to more limited access to testing among people with temporary status, differences in COVID-19 cases may be underestimated. People who are excluded from access to provincial health insurance are also excluded from this analysis. Risk of SARS-CoV-2 exposure and barriers to testing and health care may be even higher for people without health insurance in Canada, similar to uninsured people in the United States (Capps & Gelatt, 2020). However, there is no other quantitative data source that would permit analysis of COVID-19 testing and service use within this population. That administrative data capture the whole population of people registered for health insurance in BC remains a strength, and supports generalizability of findings to other Canadian jurisdictions which share the federal immigration system and similar public health insurance systems.

The data are also limited in the measures available to describe the population. Racism is a dimension of COVID-19 risk observed elsewhere that cannot be directly measured in these data (Guttmann et al., 2020; Tuyisenge & Goldenberg, 2021). We are similarly unable to directly measure living arrangements or employment, and income is a neighbourhood-level measure. People with temporary immigration status were not typically registered for the entire study period. This may partially explain the gap in access to COVID-19 testing and primary care for this group. However, given more limited access to or use of testing among people with temporary status, rates of positive SARS-CoV-2 tests may still be underreported in our data. We are missing people who registered for MSP late in the study period, as registry data were only available up to March 31, 2021. Immigration status is collected only at time of registration or renewal so may not reflect current status.

It is also necessary to recognize that the citizenship and permanent residency categories are heterogeneous, and both include people with a history of immigration. Each group contains variation in other factors that determine circumstances of increased COVID-19 risk. For example, many disabled people experienced a higher burden from COVID-19, and may be more likely to be categorized as people with citizenship in these data since people with disabilities are excluded from most immigration pathways (Pettinicchio et al., 2021). Access to safe employment may also vary markedly both within and across immigration groups. Within the category of permanent residency, there are economic and family class immigrants as well as refugees. Circumstances of COVID-19 risk and access to services may differ substantially within this group (Guttmann et al., 2020). Our analysis is not intended to examine differences between immigrants and non-immigrants defined by country of birth, but to focus on the role of immigration status held currently or at the time of health insurance registration. The ways in which immigration status shapes access to services are determined by policy and within the capacity of governments to change, which was evident during the pandemic when both Ontario and BC made temporary changes that expanded access to health care for uninsured people.

## Conclusion

People with temporary immigration status in BC experienced higher SARS-CoV-2 test positivity and lower access to testing and primary care. Interwoven immigration, health, and occupational policies place people in circumstances of higher health risk and will continue to amplify harms of the COVID-19 pandemic unless all levels of government take responsibility. Expanding past initial steps to open pathways to permanent residence and implementing the commitment to regularization for undocumented residents (Office of the Prime Minister, 2021), alongside reducing precarity of housing, income and employment associated with temporary status, and decoupling access to health care from immigration status are policies that could reduce precarity and poor health due to temporary immigration status.

## Contributions to knowledge

What does this study add to existing knowledge?

- People with temporary immigration status in BC experienced higher SARS-CoV-2 test positivity and lower access to testing and primary care.
- Test positivity varied by administrative sex, age, neighbourhood income, rurality, and health authority. Within every category of analysis, people with temporary status had the highest test positivity.

What are the key implications for public health interventions, practice, or policy?

- Expanding pathways to permanent residency to all immigrants residing in Canada may reduce the health precarity associated with temporary immigration status.
- Efforts to reduce precarity due to immigration status, including regularization pathways already under consideration, can reduce the burden of COVID-19 for both the health system and immigrants residing in BC and Canada.
- Decoupling health insurance and immigration status is needed to improve access to care for people with precarious or temporary immigration status.

**Box 1** Citizenship and permanent and temporary status in Canada

**Table:** 

Canadian citizenship* — is granted to people born within the federal borders (including Indigenous people), who go through the formal naturalization process, or to people born to or adopted by at least one parent with Canadian citizenship. People with this status have the right to enter, remain in and leave Canada, access to the full range of rights outlined in the Canadian Charter of Rights and Freedoms, and exclusively the right to vote
**Permanent residency** — is granted to people who have applied and been accepted through a variety of programs including economic class, family class, and business class immigrants, as well as resettled refugees (government assisted, privately sponsored, and blended visa office-referred), protected persons granted permanent resident status on the basis of a well-founded fear of returning to their country of origin, and successful asylum or Humanitarian and Compassionate applicants. People with permanent residency must maintain physical residency terms, and have access to most social benefits of Canadian citizens, including health care coverage, and ability to travel and work throughout Canada. They have the option of applying for Canadian citizenship after meeting length of stay, knowledge, and official language requirements
Temporary status** — describes people from other countries (“foreign nationals”) who have been authorized to be in Canada for a temporary period, and includes international students, temporary workers, visitors, refugee claimants (i.e., asylum seekers), and sponsored family members. Access to health and social services is variable and dependent on provincial, municipal, local, and individual contexts. For example, some students and workers may be eligible for provincial health insurance, refugee claimants are eligible for federal health insurance, and visitors may have private insurance or pay out of pocket for health services. Local community–organized supports, along with Community Health Centres and other clinics in various places provide health care to people regardless of immigration status

*We note this is separate from citizenship or membership in Indigenous nations, who have their own determining processes based on customary law and tradition; and, that Indigenous nations have specific legal relationships with the federal government of Canada.

**This group falls into the larger category of precarious immigration status in Canada, which includes undocumented people who are often those in the above categories who continue to live and work in Canada beyond the expiration of their temporary documentation, and whose access to health and social services is limited.

## Data Availability

The data that support the findings of this study are approved for use by data stewards and accessed through a process managed by Population Data BC. The data sets used for this study will be archived, and requests for access to them in the context of verification of study findings can be made to PopData (https://www.popdata.bc.ca/data_access). We are not permitted to share the research extract used in this analysis with other researchers.

## Appendix

Tables 4 and 5

**Table 4:** Characteristics of people who hold temporary status in British Columbia, January 2020–March 2021, *N* (%) except where indicated

	Student visa	Work permit	Refugee claimant	Convention refugee
Total number of people	108,479	97,789	2550	3397
Administrative sex				
M	55,215 (50.9)	43,522 (44.5)	1204 (47.2)	1638 (48.2)
F	53,264 (49.1)	54,267 (55.5)	1346 (52.8)	1759 (51.8)
Age				
0–19	28,545 (26.3)	183 (0.2)	816 (32.0)	876 (25.8)
20–39	77,333 (71.3)	78,207 (80.0)	948 (37.2)	1411 (41.5)
40–59	2552 (2.4)	18,678 (19.1)	712 (27.9)	905 (26.6)
60 +	49 (0.0)	721 (0.7)	74 (2.9)	205 (6.0)
Neighbourhood income quintile				
Lowest	27,826 (25.7)	26,614 (27.2)	723 (28.4)	1074 (31.6)
2nd	25,761 (23.7)	23,079 (23.6)	623 (24.4)	872 (25.7)
Middle	22,419 (20.7)	19,323 (19.8)	538 (21.1)	579 (17.0)
4th	13,923 (12.8)	14,389 (14.7)	379 (14.9)	505 (14.9)
Highest	13,420 (12.4)	11,206 (11.5)	248 (9.7)	319 (9.4)
Missing	5130 (4.7)	3178 (3.2)	39 (1.5)	48 (1.4)
Rurality				
Metropolitan	95,265 (87.8)	84,734 (86.6)	2500 (98.0)	3318 (97.7)
Small urban	9854 (9.1)	7219 (7.4)	27 (1.1)	57 (1.7)
Rural	1756 (1.6)	4539 (4.6)	10 (0.4)	8 (0.2)
Missing	1604 (1.5)	1297 (1.3)	13 (0.5)	14 (0.4)
Health authority				
Interior	9024 (8.3)	6933 (7.1)	50 (2.0)	41 (1.2)
Fraser	49,268 (45.4)	47,230 (48.3)	1682 (66.0)	2183 (64.3)
Vancouver Coastal	36,689 (33.8)	33,623 (34.4)	759 (29.8)	1036 (30.5)
Island	9212 (8.5)	6302 (6.4)	44 (1.7)	121 (3.6)
Northern	2793 (2.6)	2633 (2.7)	7 (0.3)	6 (0.2)
Missing	1493 (1.4)	1068 (1.1)	8 (0.3)	10 (0.3)

**Table 5:** Number of testing episodes (and percent positivity) by immigration status

	Citizenship	Permanent residency	Temporary status
Total testing episodes	1,808,952	364,819	69,213
Total positive	93,405 (5.2)	39,481 (10.8)	10,926 (15.8)
Administrative sex			
F	45,520 (4.6)	20,152 (9.6)	4436 (12.9)
M	47,885 (5.9)	19,329 (12.4)	6490 (18.6)
Age group			
0–19	19,792 (5.7)	2426 (12.4)	735 (13.9)
20–39	32,094 (5.4)	15,100 (9.9)	8993 (15.8)
40–59	23,914 (5.3)	15,074 (11.2)	1165 (17.8)
60 +	17,605 (4.3)	6881 (11.7)	33 (11.2)
Income quintile			
Lowest	19,217 (5.7)	10,298 (12.0)	3158 (16.5)
2nd	18,544 (5.5)	11,032 (12.5)	3346 (18.6)
Middle	19,192 (5.2)	8185 (10.9)	2229 (16.0)
4th	19,118 (4.8)	5906 (9.2)	1226 (13.1)
Highest	15,857 (4.6)	3735 (7.8)	860 (11.6)
Missing	1477 (6.7)	325 (8.2)	107 (7.6)
Rurality			
Metropolitan	72,239 (5.5)	37,604 (11.1)	10,099 (16.0)
Small urban	13,609 (4.1)	1202 (6.9)	579 (14.7)
Rural	7346 (4.4)	634 (6.9)	228 (13.1)
Missing	211 (4.7)	41 (5.5)	20 (7.8)
Health authority			
Interior	11,630 (4.2)	998 (6.9)	500 (11.9)
Fraser	49,923 (6.2)	28,853 (12.9)	7762 (20.3)
Vancouver Coastal	21,048 (5.1)	8817 (8.2)	2261 (9.9)
Island	4635 (1.9)	402 (2.7)	157 (5.4)
Northern	6081 (8.5)	400 (12.7)	232 (23.4)
